# Adipogenic Differentiation of Mesenchymal Stem Cells Alters Their Immunomodulatory Properties in a Tissue‐Specific Manner

**DOI:** 10.1002/stem.2622

**Published:** 2017-04-24

**Authors:** Hafsa Munir, Lewis S. C. Ward, Lozan Sheriff, Samuel Kemble, Saba Nayar, Francesca Barone, Gerard B. Nash, Helen M. McGettrick

**Affiliations:** ^1^ Institute for Cardiovascular Sciences College of Medical and Dental Sciences; ^2^ Institute of Inflammation and Ageing, College of Medical and Dental Sciences University of Birmingham Birmingham United Kingdom

**Keywords:** Mesenchymal stem cells, Adipocytes, Endothelial cells, Neutrophils, Inflammation, Tissue‐specific

## Abstract

Chronic inflammation is associated with formation of ectopic fat deposits that might represent damage‐induced aberrant mesenchymal stem cell (MSC) differentiation. Such deposits are associated with increased levels of inflammatory infiltrate and poor prognosis. Here we tested the hypothesis that differentiation from MSC to adipocytes in inflamed tissue might contribute to chronicity through loss of immunomodulatory function. We assessed the effects of adipogenic differentiation of MSC isolated from bone marrow or adipose tissue on their capacity to regulate neutrophil recruitment by endothelial cells and compared the differentiated cells to primary adipocytes from adipose tissue. Bone marrow derived MSC were immunosuppressive, inhibiting neutrophil recruitment to TNFα‐treated endothelial cells (EC), but MSC‐derived adipocytes were no longer able to suppress neutrophil adhesion. Changes in IL‐6 and TGFβ1 signalling appeared critical for the loss of the immunosuppressive phenotype. In contrast, native stromal cells, adipocytes derived from them, and mature adipocytes from adipose tissue were all immunoprotective. Thus disruption of normal tissue stroma homeostasis, as occurs in chronic inflammatory diseases, might drive “abnormal” adipogenesis which adversely influences the behavior of MSC and contributes to pathogenic recruitment of leukocytes. Interestingly, stromal cells programmed in native fat tissue retain an immunoprotective phenotype. Stem Cells *2017;35:1636–1646*

**Significance Statement**

Mesenchymal stem cells (MSC) can act to control inflammation, by regulating the ability of neutrophils to enter into inflamed tissues. Our data indicate that disruption of normal tissue homeostasis might drive “abnormal” MSC adipogenesis, causing the cells to lose their regulatory function. Thus, adipocytes may exist in at least two functional states: immunoprotective in healthy adipose tissue and stimulatory in sites of ectopic (e.g., chronic inflammation) fat deposition. Importantly, changes in the phenotype of MSC at sites of chronic inflammation may contribute to uncontrolled leukocyte infiltration and pathogenesis.

## Introduction

Mesenchymal stem cells (MSC) are tissue‐resident stromal precursors that undergo lineage‐specific differentiation to repair damaged tissue and modulate a variety of immune responses 1, 2. Indeed exploiting these properties therapeutically is the principle underpinning clinical trials using MSC in chronic inflammatory diseases 1. Our recent studies revealed that MSC communicate with neighboring blood vascular endothelial cells (EC) to limit leukocyte recruitment during inflammation 3, 4. This homeostatic role is a shared characteristic of MSC in a range of tissues 5. Thus, MSC act as tissue‐resident regulators of leukocyte trafficking into inflamed peripheral tissue or might be delivered therapeutically to limit acute inflammatory infiltrates or to resolve chronic inflammatory disease.

Studies of the effects of lineage‐specific differentiation on the immunoprotective properties of MSC have been rare and have yielded conflicting results [reviewed in ref. 6]. For example, human bone marrow (BM)‐derived MSC have been reported to maintain their ability to suppress the proliferation of T cells from healthy individuals or rheumatoid arthritis (RA) patients following differentiation into osteoblasts 7, 8, 9 or chrondocytes 10. Moreover, BMMSC‐derived chrondocytes reduced T‐cell activation and their ability to release proinflammatory mediators in vitro 9, 10. On the other hand, chondrogenically differentiated rat BMMSC were unable to suppress T‐cell proliferation and activation 11 or dendritic cell maturation and crosstalk with T‐cells 12. Comparable studies following adipogenic differentiation have not been reported. Thus it remains unclear whether differentiation of MSC adversely affects their immunoprotective functions, in particular their ability to regulate EC and leukocyte trafficking during inflammation.

Adipogenic differentiation of MSC is particularly interesting because ectopic fat deposits and/or alterations in local adipose tissue are associated with a number of inflammatory disorders including Duchenne muscular dystrophy 13, myocardial infarction 14, and type II diabetes 15. These deposits could be the result of inappropriate differentiation of tissue‐resident MSC, possibly induced by inflammatory mediators in the affected tissue. Indeed platelet‐derived growth factor (PDGF)receptor‐alpha positive (PDGFRα^+^) skeletal muscle MSC were identified as the source of fat deposits in a murine model of glycerol‐induced muscle fibre degeneration 13. Interestingly, cardiotoxin‐induced fibre degeneration did not lead to ectopic fat deposition in this muscle 13. These data suggest that aberrant adipogenic differentiation in peripheral tissues may be stimulus‐specific.

Chronic inflammation has been reported to alter the phenotype of BMMSC distal to the affected site. For instance, in RA and systemic lupus erythematosus, the proliferation and senescence of BMMSC were accelerated (e.g., 7, 8, 16, 17, 18), and their osteogenic capacity was reduced 19, possibly worsening disease. What changes occur to MSC locally resident in chronically inflamed peripheral tissue is uncertain, but we have shown that tissue‐resident stromal cells from chronically inflamed sites acquire a pathogenic proinflammatory phenotype, modifying EC to inappropriately recruit leukocytes 20, 21, 22, 23. The transformed stromal cells mediated their effects by altering the bioactivity of interleukin 6 (IL‐6) or transforming growth factor beta 1 (TGFβ1), switching the function of these cytokines from an immunoprotective to pro‐inflammatory state 20, 21, 23. Whether adipogenic differentiation of MSC could drive a similar proinflammatory transformation is unknown.

The foregoing led us to test the hypothesis that aberrant differentiation of MSC to adipocytes might contribute to chronicity in inflamed tissue. We assessed the effects of adipogenic differentiation of BMMSC on their capacity to regulate neutrophil recruitment during inflammation *in vitro* and compared the differentiated cells to primary adipocytes from adipose tissue. Unlike MSC, MSC‐derived adipocytes were not able to suppress neutrophil adhesion to inflamed EC. Changes in IL‐6 and TGFβ1 signalling appeared critical for the loss of immunosuppressive phenotype. In contrast, native stromal cells, adipocytes derived from them and mature adipocytes (mAD) from adipose tissue were immunoprotective. Thus disruption of normal tissue stroma homeostasis, as occurs in chronic inflammatory diseases, might drive “abnormal” adipogenesis which adversely influences the behavior of MSC and contributes to pathogenic recruitment of leukocytes.

## Materials and Methods

### Isolation and Culture of Human MSC or Adipocytes

Commercially available primary human BMMSC (Lonza Ltd., Basel, Switzerland) from healthy donors were obtained at passage 2 and expanded three times in culture (i.e., to passage 5) in Mesenchymal Stem Cell Growth Medium (MSCGM) Bulletkit (Lonza Ltd., Basel, Switzerland, http://www.lonza.com/) 3, 4, 5. Based on manufacturer's information, cells had undergone 11–12 doublings at passage 5 and underwent approximately 2.25 population doublings per passage 5.

Adipose derived mesenchymal stromal cells (ADSC; preadipocytes) or mature adipocytes (mAD) were isolated from subcutaneous adipose tissue collected from healthy patients undergoing abdominal surgery. Tissue pieces were suspended in Dulbecco's modified Eagle's medium (DMEM)/F‐12 medium (Biosera, ZI du Bousquet, France, http://www.biosera.com/) supplemented with 25 mM HEPES (Sigma‐Aldrich, Paisley, U.K., http://www.sigmaaldrich.com) containing 2 mg/ml collagenase type II (Sigma) at 37°C for 1 hour on a rotator. The cell suspension was filtered through a 70 µm pore filter and centrifuged at 400*g* for 5 minutes. Mature adipocytes floated and were harvested at the air–liquid interface, resuspended in DMEM/F‐12 medium and cultured for 24 hours prior to use. Pelleted ADSC were resuspended in DMEM/F‐12 media supplemented with 10% fetal calf serum (FCS), 100 U/ml penicillin, and 100 μg/ml streptomycin (AD basal media; all from Sigma) and cultured as previously described 24. ADSC were passaged once before use.

Alternatively commercially available primary human ADSC from healthy donors were obtained at passage 2 and expanded three times in culture (i.e., to passage 5) in MSC growth medium 2 (C‐28009; all from PromoCell GmbH, Heidelberg, Germany, http://www.promocell.com/).

### Differentiation of MSC into Adipocytes

BMMSC were cultured for 21 days in adipogenic induction medium (Lonza) according to the manufacturer's instructions and as previously described 5. To assess differentiation into MSC‐derived adipocytes, samples were fixed in 10% neutral buffered formalin (Sigma) for 30 minutes, treated with 60% isopropanol (Sigma), and stained for 30 minutes with 0.3% oil red O (Sigma) dissolved in isopropanol. Samples were washed in distilled water and counterstained with hematoxylin solution (Sigma). Cells were imaged, and digitized images were acquired using an EVOS FL Imaging System (Thermo Scientific, Loughborough, U.K., https://www.thermofisher.com/uk/en/home.html) (Supporting Information Fig. 1A).

In addition, differentiation was confirmed by the upregulation of expression of the adipocyte genes: PPARγ, C/EBPα, and FABP4. Briefly, mRNA was isolated from BMMSC and BMMSC‐derived adipocytes using the RNeasy Mini Kit (Qiagen, Crawley, U.K., http://www1.qiagen.com) 25, converted to cDNA, and analyzed by quantitative polymerase chain reaction (qPCR) using Taqman Universal PCR Mastermix according to manufacturer's instructions (Applied Biosystems, Warrington, U.K., http://www.appliedbiosystems.com) 26. Primers for PPARγ, C/EBPα, and FABP4 were bought as Assay on Demand kits from Applied Biosystems and amplified using the 7900HT Real‐Time PCR machine, analyzed using SDS 2.2 (Applied Biosystems) and expressed as 2^–ΔCT^, where C_T_ represents the difference in the cycle number between the gene of interest and 18S (Supporting Information Fig 1B–1D).

### Differentiation of Adipose Derived Preadipocytes into Adipocytes

ADSC were (5 × 10^5^) were seeded onto inverted 0.4 µm pore Transwell filter inserts (BD Biosciences, Cowley, U.K., http://www.bdbiosciences.com/eu/home) as previously described 4, 5 and cultured in AD basal media for 24 hours. Adipogenic differentiation was induced by culturing cells in DMEM/F‐12 supplemented with 166 nM recombinant insulin, 0.2 nM triiodothyronine (T3), 33 µM Biotin, 17 µM pantothenic acid, 0.01 mg/ml transferrin, and 100 nM cortisol (all from Sigma) for 21–28 days as previously described 24. For the first 5 days, media was also supplemented with 50 µg/ml 3‐isobutyl‐1‐methylxanthine and 2 µM Rosiglitazone (PPAR agonist) (all from Sigma).

### Isolation and Culture of EC

Human umbilical vein endothelial cells (HUVEC) were isolated from umbilical cords as previously described 4, 27 and cultured in Medium 199 (Life Technologies, Paisley, U.K., http://www.lifetech.com) supplemented with 20% FCS, 35 μg/ml gentamicin, 10 ng/ml epidermal growth factor, 1 μg/ml hydrocortisone (all from Sigma), and 2.5 μg/ml Amphotericin B (Life Technologies).

### Isolation of Neutrophils and Lymphocytes

Venous blood was collected from healthy donors into EDTA tubes (Sarstedt, Leicester, U.K., https://www.sarstedt.com/en/home/). Neutrophils (PMN) or peripheral blood mononuclear cells (PBMC) were isolated by centrifugation on two‐step histopaque density gradients as previously described 5, 21. PBMC were panned on culture plastic for 30 minutes to remove monocytes and purify peripheral blood lymphocytes (PBL). Purified leukocytes were washed twice in PBS containing 1 mM Ca^2+^, 0.5 mM Mg^2+^, and 0.15% bovine serum albumin (PBSA; all from Sigma) at 250*g* for 5 minutes, counted and resuspended to 2 × 10^6^ cells per ml in PBSA.

### Establishing Cocultures of EC with MSC or Adipocytes

Cocultures were established by seeding EC and stromal cells (either MSC or MSC‐derived adipocytes) on opposite sides of 0.4 µm pore Transwell filter inserts as previously described 4, 5. Briefly, MSC, MSC‐derived adipocytes, or ADSC (5 × 10^5^) were seeded onto inverted filters and cultured for 24 hours. HUVEC were then seeded on the inner surface of inserts. Cells were cocultured for 24 hours prior to treatment with or without 100 U/ml tumour necrosis factor‐α (TNFα; R&D Systems, Abingdon, U.K., http://www.rndsystems.com) for a further 4 hours (neutrophils) or TNFα in combination with 10 ng/ml interferon‐γ (IFNγ; Peprotech Inc., London, U.K., http://www.peprotech.com) for a further 24 hours (PBL) 3, 5, 25. Parallel endothelial monocultures were set up as controls.

In a separate series of experiments, nonadherent mAD (5 × 10^5^ cells) were suspended in a well, on top of which a filter was placed (such that the mAD came into contact with the basal surface of the filter). Cells were cultured 24 hours prior to seeding EC into the filter as above. Cocultures were established for 24 hours and cytokine treated as above.

In some experiments, a neutralizing antibody against IL‐6 (5 µg/ml; clone 6708; R&D Systems) was added when cocultures were established and was present throughout the coculture and cytokine stimulation.

To investigate the bioactivity of conditioned media, supernatants were obtained from MSC and MSC‐derived adipocyte monoculture and cocultures at 24 hours. Fresh EC monocultures were treated with conditioned media for 24 hours prior to stimulation with TNFα in the same conditioned media for a further 4 hours 5.

Cocultures were also formed with collagen gels containing MSC, and EC cultured on top. Rat‐tail collagen type 1 (2.15 mg/ml; First Link Ltd., West Midlands, U.K., http://www.firstlinkuk.co.uk/) was mixed with 10× M199 and then neutralized by addition of 1 N NaOH on ice, as described 21, 28, 29. MSC (2.5 × 10^4^cells per well in a 12‐well plate) were added to 500 µl, and the gel was allowed to set for 15 minutes at 37°C, and then equilibrated for 24 hours. HUVEC were seeded on the surface of the gel and cocultured with MSC for 24 hours, prior to cytokine treatment as above.

### Flow‐Based Adhesion Assay

Flow‐based adhesion assays were performed for filters incorporated into a custom‐made parallel‐plate flow chamber, using phase‐contrast digital microscopy as previously described 3, 4. Purified leukocytes were perfused over EC for 4 minutes followed by washout with cell‐free PBSA, all at a wall shear stress of 0.1 Pa. Digitized recordings of 5–10 random fields were made 2 and 9 minutes after the end of the leukocyte bolus to assess leukocyte adhesion and transmigration, respectively. Images were analyzed offline using Image‐Pro Plus software (Media Cybernetics, Marlow, U.K., http://www.mediacy.com/). Total leukocytes bound to the endothelium were counted and classified as either: (a) rolling adherent; (b) stationary, or (c) transmigrated under the endothelial monolayer. The total number of bound cells at 2 minutes was averaged per field and expressed as cells/mm^2^ per 10^6^ perfused; at 9 minutes the proportion of the bound cells that had transmigrated was evaluated.

### Collagen Gel‐Based Static Adhesion Assay

Neutrophils (5 × 10^5^ cells per ml) were added to the EC for 20 minutes, and nonadherent cells were removed by washing three times in PBSA. Neutrophil adhesion and migration was assessed using phase contrast digital microscopy by acquiring digitised z‐stack images from five random fields on the surface of the endothelium throughout the depth of the gel at 2 hours, as described 29. Images were analyzed offline using Image‐Pro Plus software. The number of neutrophils, adherent on the surface of the endothelium and throughout the gel, was averaged per field. From the known areas of the field and well, these counts were converted to totals per gel and expressed as a percentage of the number of cells added. The proportion of the adherent neutrophils migrated into the gel was also calculated.

### Gene Expression Analysis

Gene expression was analyzed using mRNA isolated from EC, MSC, and MSC‐derived adipocyte monocultures, or cocultures as described previously 5. Data were calculated as 2^‐ΔCT^ relative to expression of 18S, as described above.

### Quantification of Soluble Mediators

Culture supernatants were obtained from unstimulated EC and MSC cultured alone or in coculture for 24 hours. IL‐6 and soluble IL‐6 receptor (sIL‐6R) were quantified using IL‐6 DuoSet ELISA and sIL‐6R Quantikine ELISA Kit, respectively (R&D Systems) according to manufacturer's instructions.

### Ethics

The study was conducted in compliance with the Declaration of Helsinki. All human samples were obtained with written, informed consent and approval from the Human Biomaterial Resource Centre (Birmingham, U.K.), West Midlands and Black Country Research Ethics Committee, North East—Tyne and West South Research Ethics Committee, or University of Birmingham Local Ethical Review Committee.

### Statistical Analysis

Data are expressed as mean ± SEM, where different EC and leukocyte donors were used for each experiment. Three to ten different MSC and three primary ADSC donors were incorporated in each experiment for each independent experiment. Multivariant data were analyzed using analysis of variance (ANOVA), followed by Tukey or Dunnett post hoc tests for comparisons between treatments or to controls respectively. Univariate data were analyzed using paired *t* test. *p* ≤ 0.05 was considered statistically significant.

## Results

### Effect of Adipogenic Differentiation on the Ability of MSC to Suppress Leukocyte Recruitment by EC

**Figure 1:**
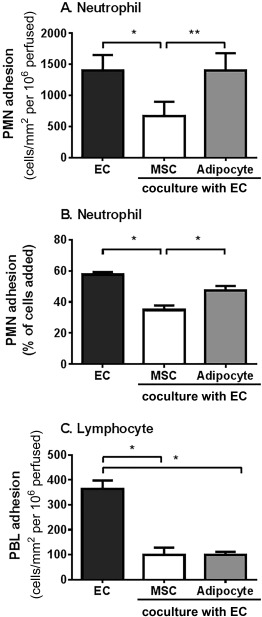
Effects of differentiation of MSC into adipocytes on their immunomodulation of neutrophil or lymphocyte recruitment. Cocultures formed by seeding EC with bone marrow MSC (BMMSC) or BMMSC‐derived adipocytes **(A, C)** on opposite sides of 0.4 µm porous filters or **(B)** seeding EC onto a collagen gel containing BMMSC or BMMSC‐derived adipocytes for 24 hours. Cultures were stimulated with (A, B) 100 U/ml tumor necrosis factor α (TNFα) for 4 hours or (C) 100 U/ml TNFα and 10 ng/ml interferon γ for 24 hours. In (A) and (C), a 4 minute bolus of purified leukocytes was perfused over the endothelium. (A) Neutrophil or (C) PBL adhesion was assessed and expressed as the number of cells adherent/mm^2^ per 10^6^ cells perfused. In (B), neutrophils were allowed to adhere to the EC cultured on collagen gel containing MSC or adipocytes for 20 minutes before adhesion was assessed at 2 hours and expressed as a percentage of cells added. Data are mean ± SEM from *n* = 3 experiments incorporating a different EC and leukocyte donor in each experiment and two different BMMSC donors. ANOVA showed a significant effect of culture conditions on neutrophil (*p* < 0.01 in A, *p* < .05 in B) and PBL adhesion (*p* < 0.01 in C). *, *p* < .05; **, *p* < 0.01 by Tukey post‐test. Abbreviations: EC, endothelial cells; MSC, mesenchymal stem cells; PBL, peripheral blood lymphocytes; PMN, neutrophil.

MSC differentiation into adipocytes was confirmed by the presence of prominent lipid droplets and the significant upregulation of the adipocyte‐related genes (PPARγ, C/EBPα, and FABP4) compared to undifferentiated MSC (Supporting Information Fig. 1). In coculture with EC, BMMSC suppressed neutrophil adhesion to TNFα‐stimulated EC under flow conditions (Fig. 1A) and in a static 3D tissue model (Fig. 1B). This effect was lost when BMMSC‐derived adipocytes were incorporated into coculture, such that they no longer inhibited neutrophil recruitment from flow (Fig. 1A) or adhesion in the 3D model (Fig. 1B). Similar effects were observed when trabecular bone or umbilical cord MSC, or MSC‐derived adipocytes were cocultured with EC (Supporting Information Fig. 2; also see Supporting information for derivation of MSC). Lymphocyte recruitment to TNFα and IFNγ‐stimulated EC was suppressed by BMMSC, but in this case, MSC‐derived adipocytes continued to suppress recruitment when incorporated into the cocultures (Fig. 1C). Adipogenic differentiation of MSC had no significant effect on the proportion of adherent leukocytes migrating through the endothelium when compared to the undifferentiated MSC cocultures or the EC cultured alone (data not shown). Thus, MSC lost their immunosuppressive effects on neutrophil, but not lymphocyte, recruitment upon differentiation into adipocytes. These responses were shared by MSC from several tissues. All subsequent experiments were performed using MSC isolated from BM.

### Role of IL‐6 in Effects of MSC and MSC‐Derived Adipocytes in Coculture

**Figure 2:**
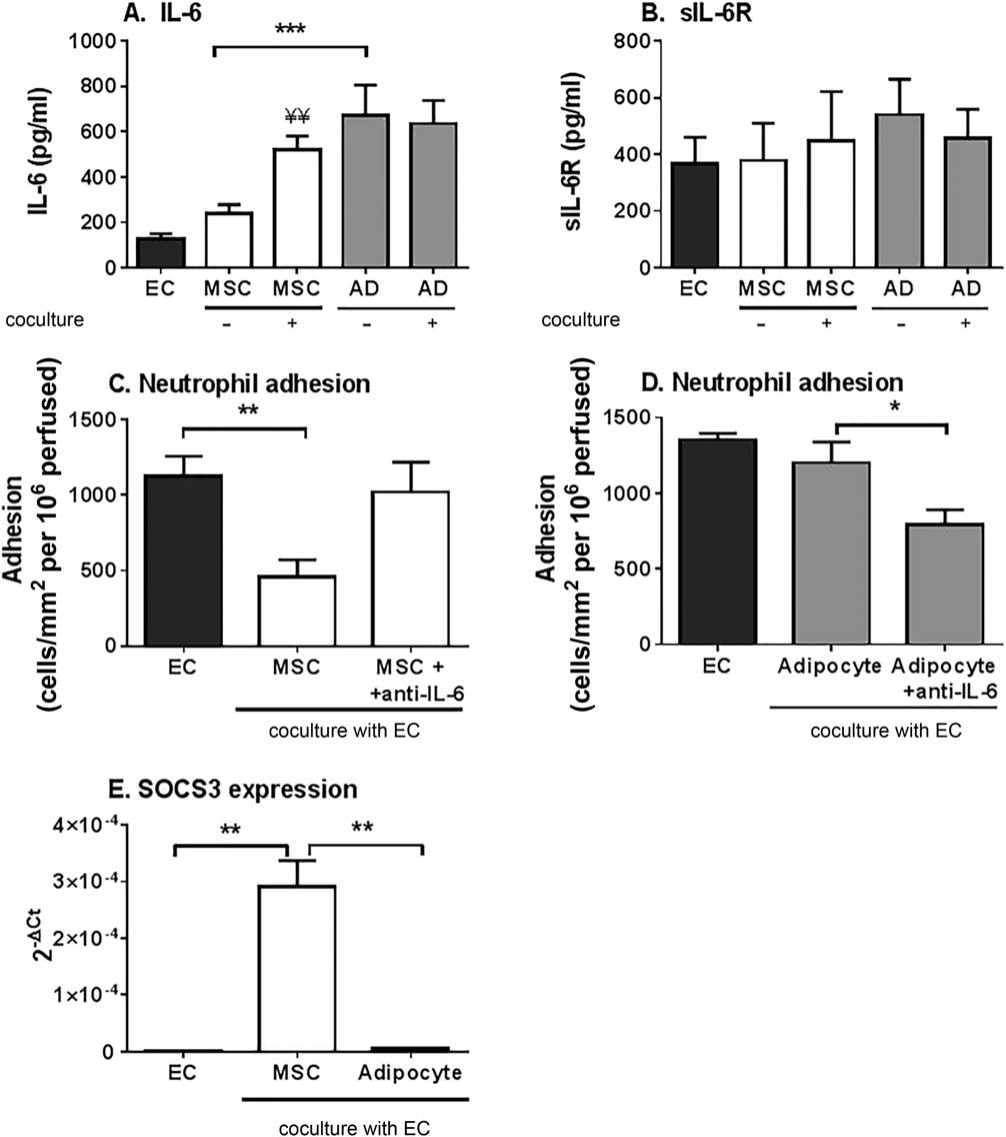
Secretion and role in immunosuppression of IL‐6 in cocultures of MSC or MSC‐derived adipocytes. **(A)** IL‐6 and **(B)** sIL‐6R release into supernatants from EC, bone marrow MSC (BMMSC), and BMMSC‐derived adipocyte monoculture and cocultures was assessed after 24 hours. **(C)** BMMSC or **(D)** BMMSC‐derived adipocyte cocultures were treated with neutralizing antibodies against IL‐6 for the duration of the coculture and cytokine treatment. Neutrophil adhesion was assessed and expressed as the number of cells adherent/mm^2^ per 10^6^ cells perfused. **(E)** SOCS3 gene expression analyzed by quantitative polymerase chain reaction in EC cultured alone, with MSC or with MSC‐derived adipocytes. Data are expressed as 2^–ΔCT^ relative to SOCS3 expression by EC cultured alone. Data are mean ± SEM (A) *n* = 16–19 and (B, C) *n* = 4, (D) *n* = 3, or (E) *n* = 3 independent experiments using a different EC and (C, D) neutrophil donor in each experiment. Three different BMMSC donors were used in all experiments, except (A) where 10 different donors were used. ANOVA showed significant effects of treatment in (A) (*p* < 0.01), (C and D) (each *p* < 0.05) *, *p* < 0.05 and **, *p* < 0.01 by Tukey post‐test. ¥¥ = *p* < 0.01 compared to the sum of the EC and respective MSC monoculture supernatant by paired *t* test. Abbreviations: AD, adipocytes; EC, endothelial cells; IL‐6, interleukin‐6; MSC, mesenchymal stem cells; sIL‐6R, soluble IL‐6 receptor; SOCS3, suppressor of cytokine signaling 3.

We have previous identified IL‐6 as the bioactive agent required for the inhibitory effects of MSC in coculture with EC 3, 5. Therefore, we compared the presence and activity of IL‐6 released in different cocultures, hypothesizing that they might account for the differences in effects of MSC and MSC‐derived adipocytes on neutrophil adhesion. MSC‐derived adipocyte monocultures released significantly more IL‐6 compared to undifferentiated MSC monocultures (Fig. 2A). MSC cocultures released a higher concentration of IL‐6 than MSC monocultures, but production of IL‐6 did not increase when MSC‐derived adipocytes were cocultured with EC (Fig. 2A). As a result, the two types of coculture generated similar levels of IL‐6. sIL‐6R levels were also similar between all culture conditions tested (Fig. 2B). Thus, loss of MSC‐mediated suppression upon adipogenic differentiation was not due to a decrease in the release of IL‐6 or its receptor in MSC‐derived adipocyte cocultures. This raised the question of whether IL‐6 present in the coculture secretome remained bioactive.

In previous studies comparing stromal cells from healthy and diseased tissues, we have reported that similar concentrations of IL‐6 produced during coculture had opposing effects (immunosuppression versus stimulation) on leukocyte recruitment 21, 30. Here we hypothesized that the differentiation of MSC into adipocytes may alter the bioactivity of IL‐6 during coculture. Neutralization of IL‐6 significantly reduced the inhibitory effects of MSC in coculture (Fig. 2C). In contrast, neutralization of IL‐6 in MSC‐derived adipocyte cocultures significantly reduced neutrophil adhesion (Fig. 2D), restoring immunoprotective function. Thus IL‐6 had essentially opposite effects in cocultures with either MSC or MSC‐derived adipocytes; supporting the concept that conversion of MSC to adipocytes resembled a pathological process.

Suppressor of cytokine signaling 3 (SOCS3) is an IL‐6 inducible gene known to regulate TNFα responses [reviewed in ref. 31]. Here, expression of SOCS3 in endothelial cells was upregulated by coculture with MSC, but not MSC‐derived adipocytes, when compared to the EC monoculture controls (Fig. 2E). Thus, downstream signaling from IL‐6/sIL‐6R differed between the two forms of coculture.

### Role of TGFβ1 Signalling in Variations in Immunosuppression

**Figure 3:**
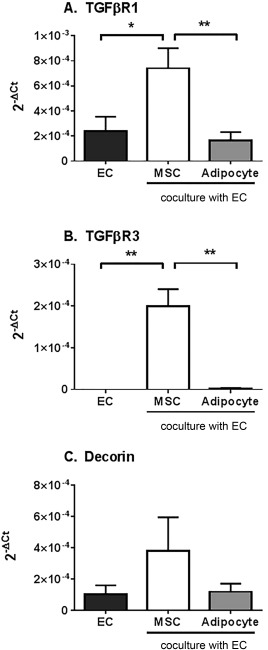
Expression and function of TGFβ receptors in endothelial cells cocultured with MSC or MSC‐derived adipocytes. Cocultures formed by seeding EC with MSC or MSC‐derived adipocytes on opposite sides of 0.4 µm porous filters for 24 hours. Gene expression for **(A)** TGFβ R1, **(B)** TGFβ R3, and **(C)** decorin was analyzed in endothelial cells cultured alone or with either MSC or MSC‐derived adipocytes by qPCR. Data are expressed as 2^–ΔCT^ relative to 18S. Data are mean ± SEM from (A) *n* = 5–6, (B) *n* = 3 or (C) *n* = 4 independent experiments using a different donor for each cell type in each experiment. ANOVA showed significant effects of treatment in (A) and (B) (both *p* < 0.01). *, *p* < 0.05 and **, *p* < 0.01 by Tukey post‐test. Abbreviations: EC, endothelial cells; MSC, mesenchymal stem cells; TGFβ, transforming growth factor beta.

We have previously reported a role for TGFβ_1_ in mediating the immunosuppressive actions of MSC in coculture 3. We wondered whether MSC differentiation might alter TGFβ_1_ responses in our cocultures. Following coculture with MSC, EC expressed significantly higher levels of TGFβ‐R_1_ and TGFβ‐R_3_ at the transcript level compared to the EC monoculture controls (Fig. 3). In contrast, MSC‐derived adipocytes had no effect on the expression of these TGFβ receptors by EC (Fig. 3). Thus loss of TGFβ_1_ downstream signaling may contribute to the changes in IL‐6 signaling and loss of immunosuppression in MSC‐derived adipocyte cocultures.

### Profile and Activity of Secretome Released by MSC and MSC‐Derived Adipocytes in Coculture

**Figure 4:**
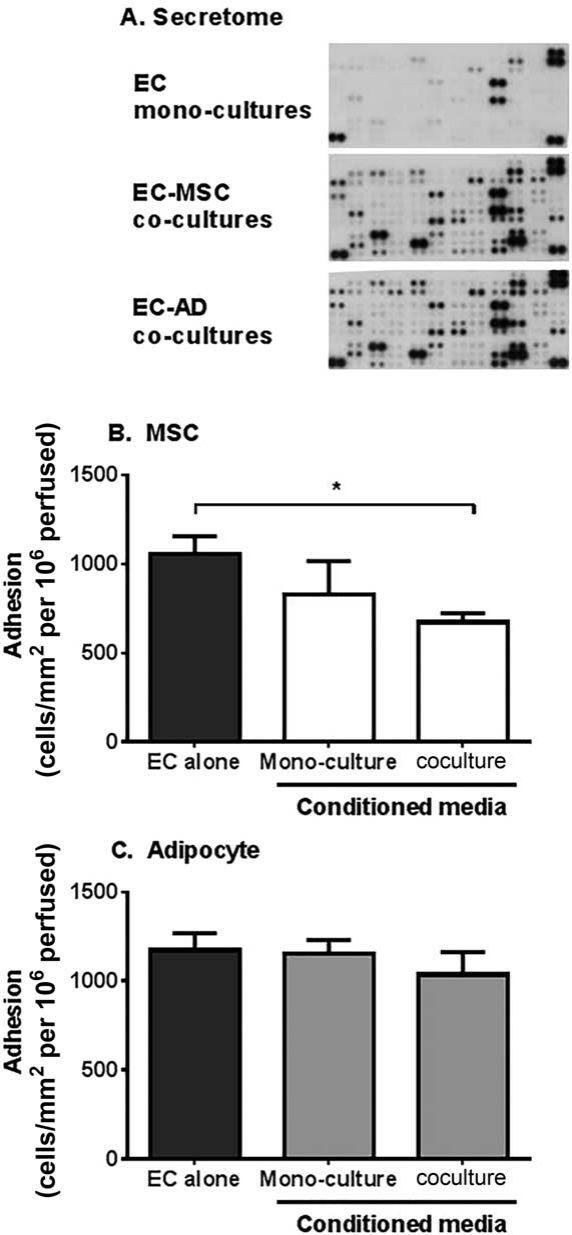
Content and immunosuppressive effects of conditioned media from MSC or adipocyte cocultures. **(A)** Conditioned media from EC alone or EC in coculture were analyzed by a cytokine expression array. Representative immunoblots from a single experiment. EC monocultures were treated with conditioned media from **(B)** BMMSC or **(C)** BMMSC‐derived adipocytes either cultured alone (monocultures) or cocultured with EC for 24 hours. Neutrophil adhesion was assessed at 2 minutes post perfusion and expressed as the number of cells adherent/mm^2^ per 10^6^ cells perfused. In (B), ANOVA showed a significant effect of treatment on neutrophil adhesion, *p* < 0.05. Data are mean ± SEM, (B) *n* = 3–5 and (C) *n* = 5 independent experiments using a different EC and neutrophil donor in each experiment. (B) 2 or (C) 3 different BMMSC donors were used. *, *p* < 0.05 by Dunnett post‐test. Abbreviations: AD, adipocytes; EC, endothelial cells; MSC, mesenchymal stem cells.

Next we examined whether differentiation into adipocytes altered the secretome generated during coculture, such that it was no longer immunosuppressive. In an unbiased analysis using a cytokine expression array (see Supporting Information for methods), we detected higher levels of some analytes (e.g., angiogenin, CD105, and platelet derived growth factor (PDGF)‐AA) and lower levels of others [e.g., Dickkopf‐related protein 1, CXCL12, vascular endothelial growth factor (VEGF)] in the MSC‐derived adipocyte coculture supernatants when compared to the MSC cocultures (Fig. 4A; Supporting Information Fig 3). In some cases, the expression of analytes, such as adiponectin, resistin, or IL‐6, was comparable between both coculture conditions (Supporting Information Fig. 3). We thus tested whether the MSC‐derived adipocyte coculture secretome had different bioactivity compared to MSC in our recruitment assays. MSC coculture conditioned media mimicked the effect of MSC‐EC coculture, inhibiting neutrophil adhesion to TNFα‐treated EC monocultures *in vitro* (Fig. 4B), as previously described 5. In contrast, conditioned media from MSC‐derived adipocyte monoculture or coculture had no effect on neutrophil adhesion (Fig. 4C). This indicates that differentiation of MSC into adipocytes alters both the composition and bioactivity of the secretome generated during coculture. Thus raises the possibility that other bioactive soluble agents generated by MSC‐derived adipocyte cocultures are capable of modulating IL‐6 and TGFβ_1_ responses, such that they are no longer immunoprotective.

### Immunosuppressive Effects of Adipocytes Derived from Adipose Tissue

**Figure 5:**
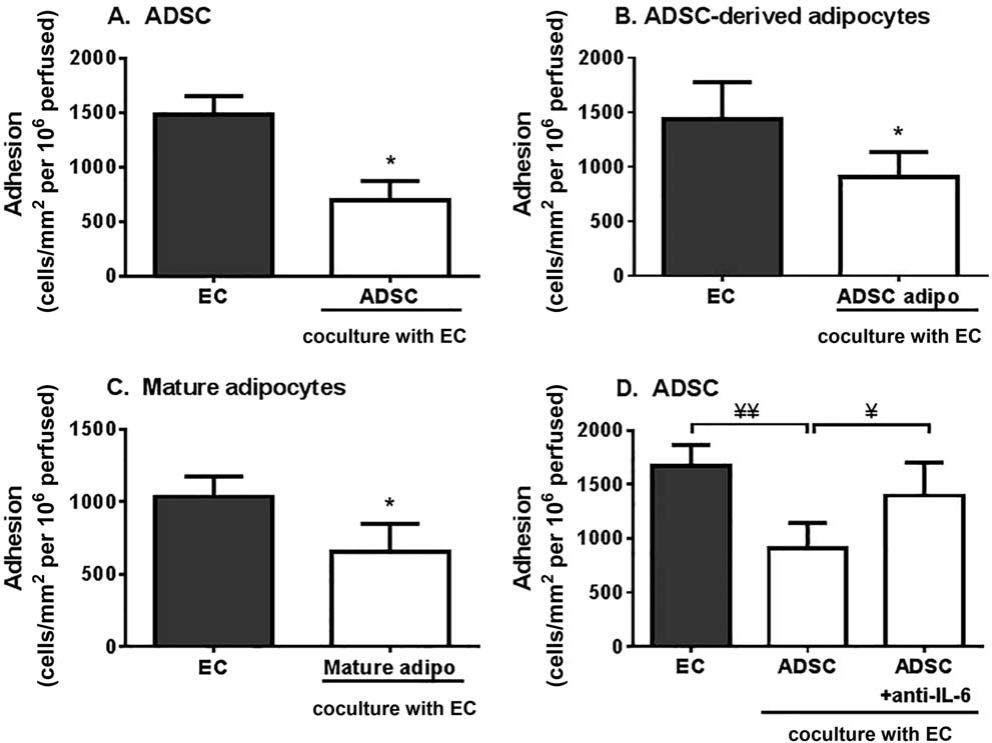
Immunosuppressive effects by ADSC and adipocytes on neutrophil recruitment. EC were cocultured with **(A)** ADSC, **(B)** ADSC‐derived adipocytes (ADSC adipo) or **(C)** mature adipocytes (Mature adipo) for 24 hours prior to stimulation with tumor necrosis factor α (TNFα) for 4 hours. **(D)** ADSC cocultures were treated with neutralizing antibodies against IL‐6 for the duration of the coculture and cytokine treatment. Neutrophil adhesion was assessed at 2 minutes postperfusion and expressed as the number of cells adherent/mm^2^ per 10^6^ cells perfused. In (D), ANOVA showed a significant effect of treatment on neutrophil adhesion, *p* < 0.05. Data are mean ± SEM, (A) *n* = 6, (B) *n* = 3, and (C, D) *n* = 4 independent experiments using a different EC and neutrophil donor in each experiment. Three different stromal cell donors were used in all experiments, except (D) where one donor was used. *, *p* < 0.05 compared to EC monoculture by paired *t* test. ¥, *p* < 0.05 and ¥¥, *p* < 0.01 by Tukey post‐test. Abbreviations: ADSC, adipose‐derived stromal cells; EC, endothelial cells; IL, interleukin.

**Figure 6:**
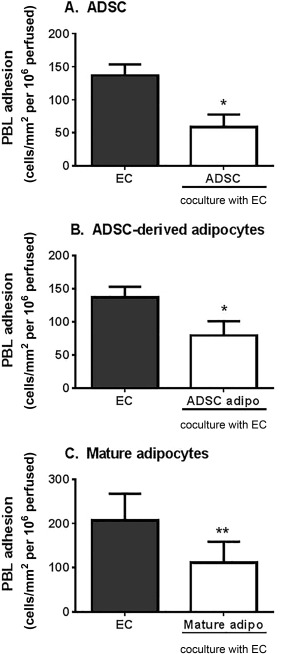
Immunosuppressive effects of ADSC and adipocytes on lymphocyte recruitment. EC were cocultured with **(A)** ADSC, **(B)** ADSC‐derived adipocytes, or **(C)** mature adipocytes (mature adipo) for 24 hours prior to stimulation with TNFα and IFNγ for 24 hours. PBL adhesion was assessed at 2 minutes postperfusion and expressed as the number of cells adherent/mm^2^ per 10^6^ cells perfused. Data are mean ± SEM from *n* = 3–4 independent experiments using a different donor for each cell type in each experiment. *, *p* < .05; **, *p* < .01 compared to EC monoculture by paired *t* test. Abbreviations: ADSC, adipose‐derived stromal cells; EC, endothelial cells; PBL, peripheral blood lymphocytes.

Finally we analyzed whether stromal cells from “true” adipose tissue (ADSC), adipocytes derived from these cells (ADSC‐derived adipocytes), and mature adipocytes (mAD) extracted from the tissue exerted the same effects as BMMSC‐derived adipocytes on leukocyte recruitment to EC. Coculture with ADSC significantly reduced neutrophil recruitment to inflamed EC (Fig. 5A), sharing the immunosuppressive capabilities seen in MSC from other tissue sources 3, 5. Unlike BMMSC‐derived adipocytes, both ADSC‐derived adipocytes and mAD were immunosuppressive in coculture, significantly inhibiting neutrophil adhesion to TNFα‐stimulated EC (Fig. 5B, 5C). Suppression of adhesion was also evident when lymphocytes were perfused over these cocultures (Fig. 6). We also detected high levels of IL‐6 in ADSC and mAD monocultures, which did not increase when these cells were cocultured with EC (Supporting Information Fig. 4). However, in contrast to BMMSC‐derived adipocytes, neutralization of IL‐6 significantly inhibited the immunosuppressive effects of ADSC in coculture (Fig. 5D). Thus adipocytes derived from adipose tissue stromal cells and “native” adipocytes maintained immunoprotective effects, while adipose cells derived from BMMSC lost this capability. It appears, therefore, that ectopic MSC‐derived adipose cells would lack the ability of 'true' adipose tissue derived stromal cells to regulate leukocyte recruitment.

## Discussion

Surprisingly little is known about how differentiation of MSC alters their immunomodulatory properties, with much of the work focusing on osteogenesis and chrondogenesis producing conflicting findings [reviewed in ref. 6]. We examined for the first time the effects of adipogenesis on MSC crosstalk with EC and regulation of the inflammatory infiltrate. We also compared this form of immunosuppression between adipocytes generated from adipose tissue, and adipocytes derived from MSC from other tissues. Interestingly, they were not the same. Upon coculture with EC, BMMSC‐derived adipocytes had lost the ability of BMMSC to suppress neutrophil adhesion. The ability to suppress lymphocyte adhesion was retained. This phenotype was shared with adipocytes differentiated from MSC derived from other non‐adipose tissue. In contrast, adipocytes differentiated from adipose‐derived stromal cells and mature adipocytes retained immunoprotective capacity, suppressing both neutrophil and lymphocyte recruitment. Thus the effect of adipogenesis on immunoregulation appears to be tissue specific, with stromal cells from adipose and nonadipose tissues generating cells with different capabilities.

A key attribute of MSC is their capacity to differentiate into various cell lineages to aid the tissue repair processes. However, we understand little about how this process affects MSC behavior, or if the context in which differentiation is triggered can alter the outcome. Here, MSC isolated from non‐adipose tissue lost some of their immunoprotective effects following adipogenesis, such that they were no longer able to inhibit neutrophil adhesion. This characteristic was not shared by ADSC, which retained their immunomodulatory capacity even after differentiation. Of note, the latter observation argues against the loss of suppression upon adipogenesis by non‐adipose‐derived MSC being an artefact of in vitro differentiation, as in vitro differentiation of ADSC had no effect on their suppressive potential.

IL‐6 is known to have different functions, eliciting immunoprotective responses or proinflammatory effects, depending on the inflammatory and stromal context [reviewed in ref. 32]. Indeed here and in our previous studies 3, 5, IL‐6 was identified as a bioactive agent required for the inhibitory effects of MSC in coculture. However, neutralization of IL‐6 reversed the effects of MSC‐derived adipocytes in coculture to be inhibitory, suggesting that under these conditions, IL‐6 had not just lost efficacy but triggered stimulatory rather than inhibitory signals. These findings are akin to those reported for synovial fibroblasts from patients with rheumatoid arthritis in coculture with EC, where IL‐6 signaling stimulated recruitment of leukocytes 20, 21. Collectively these data support the concept that conversion of MSC to adipocytes resembles a pathological process. Here, we found that in MSC cocultures, IL‐6 induced the expression of SOCS3 in EC, a typically IL‐6‐regulated gene known to downregulate cellular responses to cytokines 31, and which we previously found to play a role in modification of neutrophil recruitment by viral infection 33. Interestingly, we detected no changes in EC SOCS3 expression following coculture with MSC‐derived adipocytes, despite the presence of IL‐6 at similar concentrations as those detected in MSC cocultures. Collectively these data reveal two distinct IL‐6 signalling pathways, which are induced in a context‐specific manner and elicit different functional consequences in EC.

To the best of our knowledge only two studies have assessed MSC following adipogenesis, and neither observed any alterations in their ability to inhibit T‐cell proliferation 9 or dendritic cell maturation 12. Similarly, suppression of T‐cell proliferation remained unaffected by osteogenic differentiation of human BMMSC 7, 8, 9 or ADSC 8. On the other hand, MSC‐derived chrondocytes have been reported to inhibit 9, 10 or have no effect on 11, 12 on T‐cell responses. In the latter studies, reduction in bioactive soluble mediators, such as nitric oxide or prostaglandin E2, were linked to the defect in suppressive activity of MSC after differentiation 11. Here we also observed a different profile of soluble mediators released by MSC and differentiated MSC in coculture. Moreover, conditioned medium from MSC‐derived adipocyte cocultures was unable to suppress neutrophil recruitment in the same manner as medium generated from MSC cocultures. This clearly shows that the secretomes generated by crosstalk between EC and MSC or MSC‐derived adipocytes have different bioactivity. Thus we propose that the changes in the bioactivity of IL‐6 in MSC‐derived adipocyte cocultures occurs due to the presence of additional soluble mediators (exclusive to these cocultures) that cause a reduction in TGFβ receptors and thus response to TGFβ_1_, along with a failure to induce the suppressor genes SOCS3, usually associated with IL‐6 stimulation. Further work is required to identify the exact soluble mediator(s) responsible for these changes, which have the potential to be novel therapeutic targets for diseases involving ectopic fat deposition.

Healthy adipose tissue is considered to be intrinsically immunoprotective, largely through the abundant production of the immunosuppressive adipokine, adiponectin. Indeed our data would agree with this, with stromal cells at all stages of lineage commitment (preadipocytes, differentiated adipocytes, mAD) exhibiting immunosuppressive effects in our assays. Moreover, adipose‐derived MSC are known to inhibit T‐cell proliferation [reviewed in Ref. 34] and appear to retain this capacity after differentiation into osteoblasts 7, 8 or adipocytes 35. Thus adipocytes, much like stromal cells from other healthy tissues 5, 21, 36, have a homeostatic role in limiting inflammatory leukocyte infiltration. Indeed, IL‐6 is the shared common bioactive immunoprotective mediator of healthy stromal cells 5, 21, 36 and was responsible for the immunoprotective capacity of adipose‐derived stromal cells in coculture. Here, MSC‐derived adipocytes, ADSC, and mAD all secreted high levels of IL‐6 in culture, which were not altered by coculture with EC. Given the different effects of these cocultures, clearly it is not simply the concentration of IL‐6 but also the downstream signalling pathway(s) it elicits which are critical for its anti‐inflammatory effect. Further work is required to determine the mechanisms by which adipocytes from adipose tissue mediate their immunomodulatory effects and if these effects become modified in obesity and other metabolic disorders.

Adipogenesis in non‐adipose tissue is unlikely to be a homeostatic response, but rather the inappropriate response of the tissue to repeated episodes of damage and insult associated with chronic inflammatory diseases. Therefore, it may not be surprising that the endogenous immunoprotective effect of the tissue stroma may become distorted. Supporting this concept, neutrophil infiltration into intra‐abdominal adipose tissue was increased in mice on a high‐fat diet compared to those on a normal chow diet 37. Thus adipocytes may have the potential to exist in at least two functional states: (a) immunosuppressive as found in healthy adipose tissue and (b) stimulatory in sites of abnormal (obesity) or ectopic (chronic inflammation) fat deposition. Whether the adipocytes from the latter two sites are phenotypically and functionally the same remains to be determined. Our work also suggests an unexplored scenario; that changes in the phenotype of MSC at sites of chronic inflammation may contribute to uncontrolled leukocyte infiltration and pathogenesis.

## Conclusion

In their natural environment MSC act to endogenously dampen EC responses to cytokines, thereby limiting leukocyte recruitment in an IL‐6 dependent manner. However, aberrant differentiation of non‐adipose‐derived MSC causes the cells to lose this intrinsic immunoprotective capacity. Alterations in the phenotype and function of MSC may take the brake off and contribute to pathogenic inflammatory responses by altering the way in which IL‐6 and TGFβ_1_ exerts their effects, switching from anti‐inflammatory to pro‐inflammatory. This does not appear to be a characteristic of adipose tissue per se but may occur when MSC differentiate in an inappropriate location or circumstance.

## Author Contributions

H.M., L.W., L.S., and S.K.: collection and/or assembly of data, data analysis and interpretation, manuscript writing, and final approval of manuscript; S.N. and F.B.: provision of study material or patients, data analysis and interpretation, manuscript writing, and final approval of manuscript; G.N. and H.M.: conception and design, financial support, collection and/or assembly of data, data analysis and interpretation, manuscript writing, and final approval of manuscript.

## Disclosure of Potential Conflicts of Interest

H.M.M. has received research funding from Pfizer. All other authors indicate no potential conflicts of interest.

## Supporting information

Supporting Information
