# Supplementary material for: Adipogenic Differentiation of Mesenchymal Stem Cells Alters Their Immunomodulatory Properties in a Tissue‐Specific Manner
Source: Stem Cells. 2017 Apr 24;35(6):1636–46. doi: 10.1002/stem.2622 (PMC6052434; doi:10.1002/stem.2622)
Supplement: Supplementary file 1 — Supporting Information [file STEM-35-1636-s001.docx]

**Supplementary Information**

**Experimental Procedures**

**Isolation, culture and characterisation of human MSC**

Umbilical cord-derived MSC (UCMSC) were isolated and expanded to passage 3 as previously described (1). Briefly, blood vessels were resected and tissue pieces were suspended in PBS containing 1mg/ml collagenase type II and 1mg/ml hyaluronidase (all from Sigma) at 37^o^C on a rotator for 5h. The cell suspension was filtered and centrifuged at 400g for 5min. UCMSC were re-suspended in Low Glucose DMEM with stable L-Glutamine (Biosera) supplemented with 10% FCS, 100U/ml penicillin and 100μg/ml streptomycin (MSC medium; all from Sigma) and cultured to confluence and expanded to passage 3 (~8-9 population doublings).

Trabecular bone explants were obtained from elderly osteoarthritis patients (above the age of 60) undergoing joint replacement surgery (in collaboration with Dr Andrew Filer, University of Birmingham, UK). Bone explants were transferred to culture flasks and grown in MSC medium for 2 weeks to allow trabecular bone-derived MSC (TBMSC) to migrate away from the tissue, at which point the fragments were removed. Adherent TBMSC were then cultured to confluence and expanded to passage 3 (~10-11 population doublings).

All cultured cell populations were characterised as MSC based on the International Society for Cell Therapies criteria for defining MSC (2) as previously described (1) .

**Analysis of soluble mediators by membrane-bound cytokine expression array**

Culture supernatants were obtained from unstimulated MSC or MSC-derived adipocytes following co-culture with EC for 24h. Expression of 102 analytes (cytokines, chemokines and growth factors) were analysed using a Human XL Cytokine Array Kit (R&D Systems) according to the manufacturer’s instructions. Two technical replicates for each analyte were analysed. The membrane was imaged by X-ray, the film scanned and the average pixel density (termed integrated density) for each cytokine was analysed using ImageJ.

**Reference List**

1. Munir, H., Luu, N. T., Clarke, L. S. C., Nash, G. B., McGettrick, H. M. (2016) Comparative Ability of Mesenchymal Stromal Cells from Different Tissues to Limit Neutrophil Recruitment to Inflamed Endothelium. *PLoS ONE* 11, e0155161

2. Dominici, M., Le Blanc K, Mueller, I., Slaper-Cortenback, I., Manrini, F., Krause, D., Deans, R., Keating, A., Prockop, DJ., Horwitz, E. (2006) Minimal criteria for defining multipotent mesenchymal stromal cells. The International Society for Cellular Therapy position statement. *Cytotherapy.* 8, 315-317

**Figure Legends**

**Supplementary Fig 1: ­Characterisation of adipocytes differentiated from MSC.**

BMMSC were differentiated towards adipocyte lineage for 21 days. **(A)** Adipogenic, differentiation was assessed using Oil Red O to stain lipid droplets in **(i)** untreated BMMSC and **(ii)** differentiated BMMSC. Phase contrast and colour micrographs are representative of 3 independent experiments using 3 different BMMSC donors. Scale bar = 10µm. Gene expression for **(B)** PPARγ, **(C)** C/EBPα and **(D)** FABP4 was analysed in BMMSC and BMMSC-derived adipocytes by qPCR. Data are expressed as 2^-ΔCT^ relative to 18S. Data are mean ± SEM from n= 5 independent experiments incorporating 4 different BMMSC donors. * = p<0.05 and **= p<0.01 by paired t-test.

**Supplementary Fig 2: Effect of d**ifferentiation of TBMSC or UCMSC into adipocytes on their immunomodulation of neutrophil recruitment.

Co-cultures were formed by seeding EC with **(A)** trabecular bone (TBMSC) or **(B)** umbilical cord (UCMSC) MSC or adipocytes derived from them on opposite sides of 0.4µm porous filters for 24h prior to stimulation with TNFα for 4h. Neutrophil adhesion was expressed as a proportion of that observed on the paired EC mono-culture. In A and B, ANOVA showed a significant effect of culture conditions on neutrophil adhesion (p<0.05). Data are mean ± SEM from n=3 experiments incorporating a different EC and leukocyte donor in each and 3 different TBMSC or UBMSC donors. * = p<0.05 and ** = p<0.01 by Tukey post-test.

**Supplementary Fig 3: Content of conditioned media from MSC or adipocyte co-cultures**

Conditioned media from EC in co-culture with MSC or MSC-derived adipocytes (AD) were analysed by a cytokine expression array. The average pixel density for each cytokine was analysed using ImageJ. The integrated density of each analyte was compared between the two conditions. Representative examples show analytes that did not change (e.g. adiponectin, resistin and IL-6), were higher (e.g. angiogenin, CD105 and PDGF-AA) or lower (e.g. DKK-1, CXCL12, VEGF) in conditioned media from MSC-derived adipocyte co-cultures. Data are mean ± SEM from 2 technical replicates of each analyte from a single experiment.

**Supplementary Fig 4: IL-6 secretion by adipose-derived stromal cells and adipocytes.**

IL-6 release into supernatants from EC, ADSC and mature adipocyte (mAD) mono- and co-cultures was assessed after 24h. Data are mean ± SEM from n= 3-4 independent experiments using a different donor for each cell type in each experiment.
